# Gut Dysbiosis, Malnutrition and Sarcopenia in Liver Cirrhosis: A Narrative Review

**DOI:** 10.3390/diseases14030090

**Published:** 2026-03-02

**Authors:** Marian-Vlad Lăpădat, Claudia Georgeta Iacobescu, Ion Daniel Baboi, Maria Nedelcu, Lavinia Alice Bălăceanu, Valeria Ioana Grigorescu, Ion Dina

**Affiliations:** 1 Clinical Department 1—Medical Semiology, “Carol Davila” University of Medicine and Pharmacy, 020021 Bucharest, Romania; marian-vlad.lapadat@drd.umfcd.ro (M.-V.L.);; 2 Gastroenterology Department, Clinical Emergency Hospital “Sf. Ioan”, 042122 Bucharest, Romaniaioana-valeria.grigorescu@rez.umfcd.ro (V.I.G.); 3 Internal Medicine Department, “Carol Davila” University of Medicine and Pharmacy, 020021 Bucharest, Romania; 4 Internal Medicine Department, Clinical Emergency Hospital “Sf. Ioan”, 042122 Bucharest, Romania

**Keywords:** cirrhosis, sarcopenia, malabsorption, dysbiosis

## Abstract

Liver cirrhosis represents the end stage of chronic liver disease arising from diverse etiologies and is characterized by persistent hepatic injury, architectural distortion, extensive fibrosis, and nodular regeneration. While decompensated cirrhosis is commonly associated with overt, life-threatening complications such as hepatic encephalopathy, hepatorenal syndrome and gastrointestinal bleeding, less apparent manifestations—including sarcopenia and metabolic disturbances—have emerged as major determinants of prognosis. Sarcopenia, defined by the progressive loss of skeletal muscle mass and function, is highly prevalent in cirrhotic patients and is closely linked to frailty, increased morbidity, mortality, and adverse liver transplantation outcomes. Increasing data support the role of gastrointestinal dysfunction in the pathogenesis of sarcopenia in liver cirrhosis. In chronic liver disease, intestinal dysfunction is exacerbated by portal hypertension, which promotes increased intestinal permeability and bacterial translocation. Furthermore, gut dysbiosis, a key feature of advanced liver disease, contributes to impaired digestion, malabsorption of macro- and micronutrients, increased intestinal permeability, malnutrition and systemic inflammation. These alterations promote negative energy balance, reduce muscle protein synthesis and enhance muscle catabolism, thereby accelerating muscle wasting. Despite increasing recognition of the individual roles of gut dysbiosis, malabsorption, and sarcopenia in cirrhosis, their complex interrelationship has not been comprehensively addressed. This narrative review synthesizes current evidence on the interplay between gut dysbiosis, malabsorption and sarcopenia in patients with liver cirrhosis. We discuss underlying pathophysiological mechanisms, clinical implications and potential therapeutic strategies, while highlighting existing knowledge gaps and future research directions. Improved understanding of the gut-liver-muscle axis may offer novel opportunities for early intervention and optimization of outcomes in this high-risk patient population.

## 1. Introduction

Cirrhosis of the liver is a multifactorial and progressive disease, resulting from continuous liver damage and long-standing inflammatory responses. Overall, it is the final common pathway of diverse chronic liver conditions, such as viral hepatitis, prolonged alcohol consumption and metabolic, genetic and autoimmune liver diseases, ultimately leading to liver failure [1,2,3]. Besides well-known complications with a direct impact on survival, such as hepatic encephalopathy, hepatorenal syndrome, spontaneous bacterial peritonitis and hepatocellular carcinoma—often complicated by segmental or main portal vein thrombosis—cirrhosis is also associated with a range of metabolic disturbances that substantially contribute to disease burden [4,5,6,7]. Sarcopenia, defined by a progressive loss of skeletal muscle mass and function, represents a major complication in patients with liver cirrhosis [8]. Sarcopenia affects up to 40–70% of patients with advanced cirrhosis and is associated with poor quality of life, increased risk of complications and reduced survival [9,10,11,12,13]. Sarcopenia is closely intertwined with frailty, a multidimensional syndrome characterized by diminished physiological reserve, muscle weakness, fatigue and reduced resilience to stressors. In the context of chronic liver disease, both sarcopenia and frailty develop insidiously, often remaining underrecognized until advanced stages of cirrhosis. Numerous studies have demonstrated their strong association with increased morbidity, higher rates of cirrhosis-related complications, prolonged hospitalization and reduced quality of life [14,15,16]. While reduced dietary intake and metabolic alterations have traditionally been considered the main drivers of muscle wasting in cirrhosis, growing evidence suggests that gastrointestinal dysfunction plays a pivotal role in this process. In particular, gut dysbiosis—a hallmark of cirrhosis—has emerged as a key contributor to impaired digestion and systemic inflammation, all of which may accelerate skeletal muscle loss [13,17,18,19]. It is well established that the integrity of the intestinal barrier is essential for maintaining hepatic homeostasis, while portal hypertension resulting from advanced liver disease promotes increased gut barrier permeability, highlighting the bidirectional interactions between the gut and liver [20,21,22]. Alteration of the intestinal microbiota in liver cirrhosis occurs through two main mechanisms: a reduction in bile acid synthesis and increased portal pressure, which leads to gastrointestinal blood stasis and subsequent hyperpermeability [13]. Damage to the intestinal barrier facilitates the translocation of bacteria and microbial metabolites into the systemic circulation, resulting in endotoxemia and a proinflammatory state. This pathological condition is further amplified by the impaired capacity of the cirrhotic liver to detoxify and clear these toxins. The ensuing systemic inflammation plays a central role in the pathogenesis of sarcopenia by promoting proteolysis, suppressing muscle protein synthesis and inducing anabolic dysfunction. Moreover, chronic inflammation exacerbates metabolic alterations and energy imbalance, thereby accelerating skeletal muscle wasting and functional decline in patients with liver cirrhosis [23,24]. At the same time, an altered gut microbiota worsens disease progression through endotoxemia and the release of proinflammatory cytokines, thereby contributing to the development of cirrhosis-related complications such as hepatic encephalopathy, significant portal hypertension with esophagogastric variceal bleeding, severe hematological abnormalities, particularly thrombocytopenia and spontaneous bacterial peritonitis, ultimately creating a vicious gut–liver cycle. Malnutrition, despite being a serious complication of liver cirrhosis, is frequently underestimated. Reduced protein intake, chronic inflammation, malabsorption with altered nutrient metabolism, endocrine dysfunction and dysbiosis all contribute to the development and progression of malnutrition in cirrhotic patients [18,25,26]. Malnutrition is an independent predictor of poor clinical outcomes, including the development of complications—particularly infections—as well as increased hospitalization rates and mortality [27,28,29].

Although the individual roles of gut dysbiosis, malnutrition and sarcopenia in liver cirrhosis have been extensively discussed, their interconnection has not been comprehensively addressed in a unified narrative framework. Specifically, the mechanistic links between dysbiosis-induced malabsorption and the development of sarcopenia remain insufficiently explored. Therefore, this narrative review aims to synthesize current evidence on the interplay between gut dysbiosis, malnutrition and sarcopenia in patients with liver cirrhosis, highlighting underlying pathophysiological mechanisms, clinical implications and potential therapeutic strategies, while identifying gaps for future research.

## 2. Materials and Methods

A comprehensive literature search was conducted across major databases, including PubMed and Web of Science (Clarivate Analytics, Philadelphia, PA, USA), primarily focusing on articles published within the last five years (2020–2025), in order to capture the most recent evidence. Earlier landmark studies were also included when considered essential for understanding key pathophysiological mechanisms. The search strategy used combinations of keywords such as “liver cirrhosis,” “gut dysbiosis,” “malnutrition,” and “sarcopenia”. To minimize the risk of missing relevant studies, additional terms including “intestinal microbiota,” “gut microbiome,” “small intestinal bacterial overgrowth (SIBO),” “bacterial translocation” and “hyperammonemia” were also considered during manual reference screening and secondary searches.

Studies were selected based on their relevance to the pathophysiological links, clinical implications and management strategies related to gut dysbiosis, malnutrition and sarcopenia in patients with liver cirrhosis. Full-text original research articles, reviews and meta-analyses published in English were included, whereas case reports, editorials, and non-English publications were excluded. Data were extracted regarding study design, patient characteristics, main findings and proposed mechanisms linking gut dysbiosis, malnutrition and sarcopenia. Evidence was synthesized narratively, with emphasis on underlying mechanisms, clinical outcomes and potential therapeutic approaches. The review was conducted as a narrative synthesis and did not follow a predefined systematic review protocol. Knowledge gaps and directions for future research were highlighted. Additional relevant studies were identified through manual screening of reference lists from selected articles.

## 3. Results

The literature search identified a substantial body of evidence addressing the relationship between liver cirrhosis and sarcopenia, malnutrition and gut dysbiosis. In the Web of Science database, 586 records were identified for sarcopenia, 321 for malnutrition and 359 for gut dysbiosis, while PubMed yielded 184, 104 and 219 records for the respective topics. Following screening for relevance, the most pertinent studies were selected and synthesized narratively.

In a recent meta-analysis of 22 studies including nearly 7000 patients with liver cirrhosis, Tantai et al. reported a pooled prevalence of sarcopenia of 37.5%, with this skeletal muscle condition significantly increasing the risk of mortality. It was concluded that sarcopenia, assessed using L3-SMI criteria (skeletal muscle mass at L3 measured by computed tomography), affected approximately one-third of patients with cirrhosis, with increased prevalence reported in men, patients with alcohol-associated liver disease and those with Child–Pugh C cirrhosis [30].

According to Cui et al., sarcopenia occurred more frequently in men compared to women, and its prevalence was higher in patients with hepatic encephalopathy [31]. Specifically, patients with sarcopenia exhibited an approximately 20% higher incidence of hepatic encephalopathy compared to non-sarcopenic patients [8]. However, several studies have concluded that sarcopenia is a risk factor for hepatic encephalopathy [32,33,34].

Across different studies and meta-analyses, the reported prevalence of sarcopenia appears broadly similar, showing only slight differences [11,35,36,37], Table 1.

A prospective study including 156 patients with advanced chronic liver disease confirmed previously published findings, demonstrating a higher prevalence of sarcopenia in men, assessed using the psoas muscle index (PMI) at the level of the third lumbar vertebra on computed tomography images [38]. Furthermore, patients with alcohol-related liver disease exhibited a greater prevalence of sarcopenia than those with other causes of liver disease [38]. In addition, an association was identified between the presence of sarcopenia and greater severity of liver disease [38,39,40].

**Table 1:** Summary of the main studies evaluating the prevalence and clinical impact of sarcopenia in patients with liver cirrhosis.

Author (Year)	Study Design	Population/Sample Size	Sarcopenia Prevalence	Main Findings
Tantai X et al. (2022) [30]	Meta-analysis	6965	37.50%	Independent risk factor for mortality
Cui Y et al. (2024) [31]	Systematic review/Meta-analysis	8945	41%	Risk factor for both survival and mortality; significant risk factor for HE *.
Mazeaud S et al. (2023) [35]	Systematic review/Meta-analysis	8821	33%	Indicator of poor quality of life
Thyloor Kenchappa S (2023) [38]	Prospective Study	156	47.40%	Indicator of worse prognosis, increased hospitalization and mortality rate
Tuo S et al. (2024) [41]	Systematic review/Meta-analysis	13,158	40.10%	Independent predictor of poor quality of life, increased morbidity and mortality.

* HE—Hepatic Encephalopathy.

A large systematic review and meta-analysis encompassing 55 studies with a total of 13,158 patients across 17 countries reported an overall worldwide prevalence of sarcopenia of 40.1% and identified sarcopenia as an independent predictor of prolonged hospital stays, poor quality of life, increased morbidity and mortality in cirrhotic patients [41].

Sarcopenia and malnutrition are closely interrelated in patients with liver cirrhosis, with sarcopenia often reflecting the clinical manifestation of nutritional deficiencies and contributing to increased morbidity and mortality. Malnutrition in cirrhotic patients has been reported in 5–92% of cases, highlighting either inconsistencies in assessment methods, limited clinical awareness or both factors [18].

Several authors have investigated different instruments and methods for assessing malnutrition in patients with liver cirrhosis due to the lack of consensus regarding its diagnosis, noting that malnutrition is often underdiagnosed in clinical practice [42]. They recommended the use of anthropometric tools, such as triceps skinfold thickness (TST) for assessing fat mass in females and mid-arm muscle circumference (MAMC) for detecting muscle loss in males. These tools have been employed in multiple studies to identify malnutrition, with TST demonstrating a detection range of 10–45%; moreover, reduced TST measurements have been associated with increased mortality [43,44,45,46,47].

An extensive review regarding the molecular mechanism of malnutrition published in 2020 outlined established and emerging molecular mechanisms involved in the development of malnutrition and sarcopenia in liver cirrhosis and identifies key gaps in current knowledge [48].

All of these are summarized in Table 2.

Beyond sarcopenia and malnutrition, gut dysbiosis has emerged as an important contributor to disease progression in liver cirrhosis. Liver–gut axis has recently gained considerable research interest, with numerous publications highlighting its contribution to gut dysbiosis, systemic inflammation and metabolic alterations in patients with liver cirrhosis, Table 3.

Nie et al. described the potential mechanisms underlying gut dysbiosis in liver cirrhosis and examined the development of related complications, including spontaneous bacterial peritonitis, hepatorenal syndrome, portal vein thrombosis, hepatic encephalopathy and hepatocellular carcinoma [13].

The composition of the gut microbiota is critically important, as highlighted by a recent large meta-analysis [49]. Alterations associated with liver cirrhosis were characterized by a reduction in *Lachnospiraceae*, *Ruminococcaceae* and *Clostridia*, with an increase in *Enterobacteriaceae*, *Pasteurellaceae*, *Streptococcaceae* and *Streptococcus* species [49].

Another meta-analysis including 17 relevant studies confirmed previously reported data from literature regarding gut microbiota composition in patients with liver cirrhosis [50]. Cirrhosis is associated with reduced levels of *Lactobacillus* and *Bifidobacterium*, along with an overrepresentation of *Enterobacter* and *Enterococcus*. Probiotic-based interventions, particularly those containing *Bifidobacterium* alone or in combination with *Lactobacillus*, have been shown to lower blood ammonia concentrations and decrease the risk of hepatic encephalopathy [50,55,56,57].

An original study, although conducted in a limited number of subjects, confirmed the association between gut microbiota alterations and an increased risk of complications in liver cirrhosis; in particular, these changes correlated with disease severity [51].

The authors of a meta-analysis showed that the gut microbiota is different in liver cirrhosis with hepatic encephalopathy versus without hepatic encephalopathy [52].

A large meta-analysis including 17 studies revealed characteristic alterations in the gut microbiota of patients with liver cirrhosis and hepatic encephalopathy which may predict the development of encephalopathy and overall prognosis [53].

Another meta-analysis including 30 randomized controlled trials demonstrated promising results regarding the use of probiotics in reversing the manifestations of hepatic encephalopathy by reducing ammonia levels and improving neuropsychometric and neurophysiological performance. In addition, probiotics were shown to potentially enhance liver function, as reflected by a significant reduction in MELD score [54]. Furthermore, probiotic therapy has been investigated across various liver diseases, including liver cirrhosis with hepatic encephalopathy and non-alcoholic liver disease, with encouraging outcomes [58,59,60].

## 4. Discussion

### 4.1. Clinical Relevance of Sarcopenia and Malnutrition in Liver Cirrhosis

Historically, sarcopenia was first proposed by Rosenberg in 1989 and originally referred solely to the age-related loss of skeletal muscle mass, termed primary sarcopenia disease [61,62,63]. However, the concept has since evolved to encompass additional features, including declines in muscle strength and physical performance, which are classified as secondary sarcopenia when related to an underlying disease [64,65,66]. In this context, sarcopenia and malnutrition have emerged as highly prevalent and clinically relevant complications of liver cirrhosis, with consistent evidence demonstrating their strong association with increased morbidity, mortality and reduced quality of life. Sarcopenia tends to become more prevalent as liver disease severity increases, as reflected by worsening Child–Pugh class [11,12,67]. As liver disease progresses, the increasing prevalence of sarcopenia substantially contributes to the development of frailty, a multidimensional clinical syndrome characterized by reduced functional capacity, muscle impairment and decreased tolerance to physical and metabolic challenges. Importantly, sarcopenia and frailty are increasingly recognized as key determinants of adverse clinical outcomes in patients with liver cirrhosis, with sarcopenia independently predicting increased mortality [15,16,30]. Beyond a low dietary intake and metabolic alterations, emerging evidence highlights gastrointestinal dysfunction, particularly gut dysbiosis, as a central contributor to sarcopenia in cirrhosis. Dysbiosis promotes systemic inflammation and nutrient malabsorption, thereby accelerating muscle wasting and exacerbating frailty. Accurate assessment of skeletal muscle mass using L3-SMI allows for objective identification of sarcopenic patients, facilitating risk stratification and guiding clinical management in cirrhosis [14,68].

### 4.2. Pathophysiological Interplay Between Gut Dysbiosis, Malnutrition and Sarcopenia

The gut–liver–muscle axis represents a central mechanism linking gastrointestinal dysfunction, malnutrition and sarcopenia in patients with liver cirrhosis. Alterations in gut microbiota composition, commonly observed in advanced liver disease, are characterized by reduced beneficial bacteria such as *Lactobacillus* and *Bifidobacterium*, alongside overrepresentation of potentially pathogenic taxa including *Enterobacter* and *Enterococcus* [49,50,52,53]. These microbial imbalances contribute to increased intestinal permeability, bacterial translocation and systemic inflammation, which impair nutrient absorption and metabolism [21]. Consequently, a negative energy balance develops, reducing muscle protein synthesis and promoting catabolic pathways, thereby accelerating skeletal muscle loss. Malnutrition in cirrhotic patients is multifactorial, encompassing inadequate dietary intake, altered macronutrient utilization and micronutrient deficiencies, often exacerbated by dysbiosis. Evidence suggests that the severity of gut microbiota alterations correlates with the progression of liver disease and the risk of complications, including hepatic encephalopathy, reinforcing the clinical importance of maintaining gut microbial homeostasis [18,69,70,71]. Beyond its musculoskeletal consequences, gut dysbiosis contributes to worsening portal hypertension, which in turn can lead to several complications, such as esophagogastric varices with risk of bleeding, ascites, spontaneous bacterial peritonitis, hypersplenism with thrombocytopenia and portosystemic encephalopathy [72]. On the other hand, it has been hypothesized that portal hypertension, by impairing intestinal motility and barrier function, promotes cirrhosis-specific gut dysbiosis, reinforcing the vicious gut–liver–cycle [73]. Endotoxemia and systemic inflammation arising from gut dysbiosis further exacerbate liver dysfunction and increase portal pressure, contributing to complications such as esophagogastric variceal bleeding and hypersplenism-related thrombocytopenia. Thrombocytopenia reflects both splenic sequestration and bone marrow suppression, complicating invasive procedures and overall management. Partial splenic embolization has emerged as an effective strategy to restore platelet counts and reduce bleeding risk [74]. Importantly, despite the biological plausibility of this integrated model, direct clinical evidence specifically linking microbiota composition with objectively measured sarcopenia in cirrhosis remains scarce, highlighting the need for dedicated translational studies.

### 4.3. Role of Inflammation, Metabolic Dysregulation and Ammonia

Systemic inflammation represents a central pathophysiological mechanism between gut dysbiosis and muscle wasting in cirrhosis [13]. Elevated circulating endotoxins and proinflammatory cytokines contribute to anabolic resistance, mitochondrial dysfunction and impaired energy metabolism in skeletal muscle [21,23,75,76,77]. In parallel, hyperammonemia—partly driven by dysbiosis—has been shown to directly impair muscle protein synthesis and promote myostatin expression, further accelerating sarcopenia [78,79,80]. These mechanisms also help explain the close association between sarcopenia and hepatic encephalopathy, as skeletal muscle plays a compensatory role in ammonia detoxification. Loss of muscle mass therefore exacerbates hyperammonemia and neurocognitive dysfunction, reinforcing the bidirectional relationship between muscle wasting and hepatic encephalopathy. Beyond their metabolic and neuromuscular consequences, the same systemic inflammatory and metabolic derangements also promote endothelial dysfunction and coagulation abnormalities in advanced cirrhosis, highlighting the potential role of D-dimers as biomarkers of disease severity [81,82].

### 4.4. Prognostic and Therapeutic Implications

The close interplay between gut dysbiosis, malnutrition, sarcopenia and systemic inflammation have important implications for prognosis and clinical management in patients with liver cirrhosis. Increasing evidence indicates that sarcopenia and frailty are not merely consequences of advanced liver disease but represent independent predictors of adverse outcomes, including mortality, risk of decompensation and reduced quality of life [30,31,49]. Sarcopenia has been consistently associated with a higher incidence of cirrhosis-related complications, including infections, hepatic encephalopathy, variceal bleeding and prolonged hospitalization [9,11]. Sarcopenia and frailty provide complementary prognostic information and should be systematically assessed together in routine clinical practice.

These considerations are particularly relevant in the context of liver transplantation because both sarcopenia and frailty have been linked to increased waitlist mortality, lower likelihood of successful transplantation and worse post-transplant outcomes. Consequently, objective evaluation of skeletal muscle mass is increasingly advocated as part of pre-transplant risk stratification. The third lumbar skeletal muscle index (L3 SMI) was a more accurate predictor of mortality than the psoas muscle index (PMI) [83,84]. From a therapeutic perspective, recognition of the gut–liver–muscle axis supports the implementation of integrated, multimodal intervention strategies. Nutritional therapy remains the cornerstone of sarcopenia management in cirrhosis and includes adequate protein intake, late-evening snacks, correction of micronutrient deficiencies, while routine branched-chain amino acid supplementation beyond meeting daily protein targets from diverse sources is not recommended [84]. However, nutritional interventions alone may be insufficient in the presence of persistent inflammation and gastrointestinal dysfunction.

Targeted modulation of the gut microbiota has therefore emerged as a promising adjunctive strategy. Probiotics, prebiotics and synbiotics have been shown to improve gut barrier integrity, reduce endotoxemia, lower ammonia levels and decrease the incidence of hepatic encephalopathy, with potential improvements in liver function, as reflected by reductions in MELD score [54]. By attenuating systemic inflammation and improving nutrient absorption, microbiota-directed therapies may contribute to the preservation of muscle mass and functional status; however, further high-quality studies are needed to define optimal regimens. Despite these encouraging findings, heterogeneity in probiotic strains, dosing and study design limits firm clinical recommendations [58,59].

Exercise-based interventions, when appropriately tailored to disease severity, represent an additional pillar of multimodal management. Resistance and aerobic training have been shown to improve muscle strength, physical performance and quality of life in selected patients with cirrhosis. When combined with nutritional and microbiota-targeted therapies, these interventions form the basis of a “prehabilitation” strategy, particularly relevant for patients awaiting liver transplantation [84,85].

### 4.5. Critical Appraisal of Current Evidence

A substantial body of literature has addressed gut dysbiosis and sarcopenia in liver cirrhosis; however, important limitations remain in the current evidence base. To date, very few studies have been specifically designed to directly investigate the association between gut microbiota alterations and objectively defined sarcopenia as a primary outcome [13,55]. Most available data derive from investigations that examined gut dysbiosis in relation to hepatic decompensation or hepatic encephalopathy, while sarcopenia has predominantly been evaluated as a prognostic factor for mortality, hospitalization and complications [13,30,31,41,53]. Therefore, the proposed dysbiosis–sarcopenia link is largely constructed from indirect associations rather than demonstrated causal relationships.

In addition, cirrhosis represents a highly heterogeneous clinical entity, spanning from compensated stages to advanced decompensated disease characterized by portal hypertension, systemic inflammation and multiorgan dysfunction [1,4]. Both dysbiosis and sarcopenia appear to worsen with increasing disease severity [30,49]; however, most studies do not consistently stratify outcomes according to compensated versus decompensated cirrhosis [71]. This methodological limitation restricts the ability to determine whether gut microbiota alterations directly contribute to early muscle loss or whether both conditions primarily reflect advanced hepatic insufficiency.

Hyperammonemia illustrates the complexity of this bidirectional interplay. Ammonia accumulation results from impaired hepatic detoxification, portosystemic shunting and dysbiosis-driven intestinal ammonia production [78,79]. Elevated ammonia levels impair skeletal muscle protein synthesis and promote myostatin expression via NF-κappaB-mediated mechanisms [80]. Conversely, skeletal muscle functions as an auxiliary site for ammonia detoxification; consequently, sarcopenia further aggravates hyperammonemia, creating a self-perpetuating cycle [8,32]. Importantly, much of the mechanistic evidence supporting this model derives from experimental studies or indirect clinical observations rather than from prospective human studies specifically designed to assess the gut–liver–muscle axis.

These considerations highlight the need for integrated longitudinal studies combining microbiome profiling, objective muscle mass quantification and metabolic characterization across clearly defined stages of cirrhosis.

### 4.6. Knowledge Gaps and Future Directions

Despite growing recognition of the clinical relevance of sarcopenia and frailty in chronic liver disease, several important knowledge gaps remain. Standardized diagnostic criteria and cut-off values applicable across diverse populations and disease stages are still lacking, limiting comparability between studies and routine clinical implementation. In addition, the optimal integration of sarcopenia and frailty assessments into existing prognostic models requires further validation. From a therapeutic perspective, high-quality randomized controlled trials are needed to define the most effective nutritional strategies, exercise interventions and microbiota-targeted therapies, as well as their timing and duration. The role of emerging pharmacological agents and personalized interventions based on sex, age, disease severity and comorbidities also warrants further investigation. Future research should focus on longitudinal studies to clarify causal relationships, evaluate clinically meaningful outcomes and determine whether targeted interventions can improve survival, functional status and quality of life. Advances in imaging, digital health tools and biomarker discovery may further enhance risk stratification and enable individualized management strategies. Finally, emerging biomarkers reflecting systemic inflammation, metabolic dysregulation and coagulation imbalance, such as ammonia levels and D-dimers, may further refine prognostic assessment and guide individualized therapeutic approaches. As discussed earlier, these findings emphasize the need for early identification and comprehensive management of sarcopenia, malnutrition and gut dysbiosis to improve outcomes in patients with liver cirrhosis.

In addition to nutritional optimization and microbiota-targeted interventions, structured liver rehabilitation programs have recently emerged as a promising therapeutic strategy in chronic liver disease. Liver rehabilitation integrates individualized exercise training, nutritional support and multidisciplinary care with the aim of improving physical function, attenuating sarcopenia and enhancing overall clinical outcomes. Recent guidance by Terai et al. emphasizes the importance of tailored rehabilitation protocols according to disease severity, functional status and comorbidities, highlighting their potential role in preventing frailty progression and improving quality of life in patients with advanced liver disease. Incorporating standardized rehabilitation strategies into routine cirrhosis management may therefore represent an important step toward comprehensive, multimodal care, although further prospective studies are required to define optimal timing, intensity and long-term benefits [86].

## 5. Conclusions

Sarcopenia, malnutrition and gut dysbiosis are highly prevalent and interrelated complications of liver cirrhosis, significantly impacting prognosis and clinical outcomes. The gut–liver–muscle axis plays a central role in disease progression through systemic inflammation, metabolic dysregulation and impaired nutrient absorption. Early recognition and integrated, multimodal management strategies targeting nutrition, physical function and gut microbiota are essential to ameliorate health status in patients with liver cirrhosis.

Importantly, the recognition of the gut-liver-muscle axis reframes liver cirrhosis as a systemic disorder extending far beyond hepatic insufficiency alone. Muscle depletion should not be viewed merely as a secondary epiphenomenon of advanced disease but as an active contributor to metabolic instability, ammonia handling and overall clinical deterioration. Early identification of sarcopenia, particularly in compensated stages of cirrhosis, may represent a critical window for intervention before the establishment of irreversible frailty and recurrent decompensation. In this context, integrated management strategies that combine optimized protein intake, structured physical rehabilitation and microbiota-targeted therapies may provide synergistic benefits.

Furthermore, future research should prioritize longitudinal, well-characterized cohorts in which microbiome profiling, objective muscle mass quantification and metabolic biomarkers are assessed simultaneously across different stages of cirrhosis. Such approaches could clarify causality, identify high-risk phenotypes and facilitate personalized therapeutic strategies. Ultimately, embedding muscle health and intestinal homeostasis into standard cirrhosis care pathways may improve transplant candidacy, reduce healthcare utilization and enhance long-term survival in this vulnerable population.

## Figures and Tables

**Table 2:** Summary of the main studies evaluating the prevalence and clinical impact of malnutrition in patients with liver cirrhosis.

Author (Year)	Study Design	Prevalence	Mechanisms/Diagnosis	Main Outcomes
Traub J et al. (2021) [18]	Review	5–92%	Inflammation, malabsorption, reduced protein intake, dysbiosis, hormonal disturbances	Risk factor for infections and HE
Bartlett S. et al. (2025) [42]	Narrative Review	40–75%	No correlation with biochemical markers	Increased rates of complications, hospitalization and mortality
Meyer F. et al. (2020) [48]	Review	50–90%	No validated biomarkers for identification and monitoring of DRM *	Skeletal muscle loss is the principal component of malnutrition

* DRM—disease-related malnutrition.

**Table 3:** Summary of the main studies evaluating the clinical impact of dysbiosis in patients with liver cirrhosis.

Author (Year)	Study Design	Population/Sample Size/Studies	Microbiota Components	Main Outcomes
Liu Y et al. (2025) [49]	Systematic Review/Meta-analysis	5076 subjects	Increase *Enterobacteriaceae* and *Pasteurellaceae*	Gut microbiota imbalance plays a key role in liver cirrhosis progression.
Huang L et al. (2022) [50]	Systematic Review/Meta-analysis	17 studies	Decrease *Bifidobacterium*, *Lactobacillus*	Targeted probiotic supplementation reduce * HE incidence
Solé C et al. (2021) [51]	Original Research	182 subjects	Reduction metagenomic species richness	Altered microbioma increased the risk of cirrhosis complications/survival.
Xirouchakis E et al. (2025) [52]	Systematic Review	9763 subjects	Increase *Enterococcus*, *Streptococcus*	Dysbiosis associated with * HE and ** HCC
Xu XT et al. (2025) [53]	Systematic Review/Meta-analysis	17 studies	In HE, increase *Enterococcus*, *Proteobacteria*, *Enterobacteriaceae*	The gut microbiome may distinguish between patients with/without HE in liver cirrhosis
Yang X et al. (2024) [54]	Systematic Review/Meta-analysis	30 *** RCTs/2084 subjects	NA	Probiotics may reverse HE, regulate gut dysbiosis, increase quality of life

* HE—hepatic encephalopathy; ** HCC—hepatocellular carcinoma; *** RCTs—randomized controlled trial.

## Data Availability

No new data were created or analyzed in this study. Data sharing is not applicable to this article.
